# The Role of Pretreatment Neutrophil to Lymphocyte Ratio and Platelet to Lymphocyte Ratio in the Diagnosis of Breast Cancer: Predicting Lymph Node Metastasis

**DOI:** 10.4021/wjon744w

**Published:** 2014-01-16

**Authors:** Aydan Eroglu, Serkan Akbulut

**Affiliations:** a Department of General Surgery, Surgical Oncology Unite, Ankara University Medical School, Cebeci Kampus, Dikimevi, Ankara 06580, Turkey

## To the Editor

Clinical outcome in cancer patients can be determined by the host-response factors as well as the tumor characteristics. In the last decade, the markers of systemic inflammatory response including neutrophil, platelet and lymphocyte have been investigated in various types of malignant tumors [1]. Cancer development enhances chronic inflammation. On the other hand, the inflammation can enhance tumor growth, invasion and metastasis. Although the elevated neutrophil counts can be related to paraneoplastic activity of the tumor, the low lymphocyte counts can be associated with immune system suppression in cancer. Both the increased neutrophil and platelet, and conversely, decreased lymphocyte can be associated with poor prognosis. In addition, the increased neutrophil to lymphocyte ratio (NLR) in cancer patients can be related to the limited immune capability during carcinogenesis. One of the routinely available markers of systemic inflammatory response is NLR, which is derived from absolute neutrophil and absolute lymphocyte counts of the full blood count. The routine preoperative platelet to lymphocyte ratio (PLR) is also readily available laboratory variable without additional costs.

Some studies have shown that patients with higher NLR have significantly lower survival rates than those with lower NLR in different cancer types including breast cancer. Recently, only two studies by Azab et al and Noh et al have been evaluated if NLR is predictive of breast cancer-specific mortality or disease-free survival [2, 3]. The other recent report by Seretis et al has shown that the mean NLR was significantly elevated in thyroid cancer compared with benign thyroid disorders [4]. Besides, Azab and coworkers comparing the prognostic value of NLR versus PLR in breast cancer have shown the NLR to be superior in predicting long-term breast cancer specific mortality [3]. However, to our best knowledge, the usefulness of pretreatment NLR and PLR to diagnosis in breast cancer has not been reported. Encouraged by the studies, we have retrieved data in breast cancer as well as benign breast lesions and performed an analysis to assess the usefulness of pretreatment NLR and PLR with respect to the diagnosis of breast cancer. We have also investigated if the NLR and PLR are associated with the lymph node metastasis.

The study consisted of 60 breast cancer patients (group 1) and 35 cases with benign breast lesions including fibrocystic changes, sclerosing adenosis and fibroadenoma (group 2).

Patients with active infection, any known other cancer history, hematologic disorders, chronic or current steroid treatment, and chronic inflammatory or autoimmune disorders were excluded from sample groups. The pretreatment NLR and PLR were calculated by total neutrophil or platelet counts divided by total lymphocyte number.

Results were expressed as mean values ± standard deviation with range and were compared using Student’s t test for two groups or ANOVA test for more than two groups. A P value < 0.05 was considered statistically significant. All the statistical analyses were conducted using SPSS version 15.0 (SPSS Inc., Chicago, IL, USA).

The mean pretreatment NLRs were 2.53 ± 1.4 and 2.08 ± 0.7 in group 1 and group 2, respectively (P = 0.045). No difference was observed in the distribution of PLR in breast cancer patients versus patients with benign lesions (P > 0.05) (Table 1). Breast cancer group was also divided into two groups according to lymph node metastases. Among 60 breast cancer patients, 34 patients had lymph node metastases. The mean NLRs were 2.87 ± 1.5 and 2.08 ± 0.8 in patients with or without lymph node metastasis, respectively (P = 0.017). An elevated level of PLR in patient with lymph node metastasis was found, but this did not reach statistical significance (P = 0.076).

**Table 1:** The Distribution of the Mean of NLR and PLR According to the Groups

Variable	NLR*	P value	PLR*	P value
Groups				
Group 1 (n = 60)	2.53 ± 1.3	0.045	148.5 ± 56	0.408
Group 2 (n = 35)	2.08 ± 0.7		138.4 ± 55	
Lymph node metastasis				
Yes (n = 34)	2.87 ± 1.5	0.017	156.5 ± 56	0.076
No (n = 26)	2.08 ± 0.8		138.4 ± 55	

NLR: neutrophil/lymphocyte ratio; PLR: platelet/lymphocyte ratio. Group 1, breast cancer patients; Group 2, benign breast tumor. n: number of cases; *: the mean ± standard deviation.

The tumor/host interaction may have important influence on carcinogenesis. Cancer-associated inflammation is also an important determinant of outcome in cancer patients. Although recent studies have reported that high level of NLR is an independent risk factor for poor prognosis in patients with various types of cancer including breast cancer [1-3], there are limited data related to NLR or PLR as potential marker in the context of malignancy diagnosis. As far as we know, the presented report was the first study that has investigated the possible correlations of pretreatment NLR and PLR in breast cancer and benign breast lesions. However, there are some limitations of the study. First, we did not determine NLR and PLR in healthy women. Second limitation was the small sample size of patients. Further studies with larger series are necessary to confirm our preliminary results. Our findings support the hypothesis that high level of NLR can influence the lymph node metastasis in breast cancer. However, further studies are required to better understand the role of NLR value in predicting lymph node metastasis, in particular, during sentinel lymph node biopsy before a conclusion can be drawn.
